# Gaps in Migrants’ Access to Contraceptive Services: A Survey of Nepalese Women and Men in Japan

**DOI:** 10.3390/healthcare12131320

**Published:** 2024-07-02

**Authors:** Masako Tanaka, Rachana Manandhar Shrestha, Richa Shah, Divya Bhandari, Bijay Gyawali

**Affiliations:** 1 Faculty of Global Studies, Sophia University, Tokyo 102-8554, Japan; 2 Institute of Asian, African, and Middel Eastern Studies, Sophia University, Tokyo 102-8554, Japan; rach.manandhar@gmail.com; 3 Department of Community and Global Health, The University of Tokyo, Tokyo 113-8654, Japan; 4 Health Action and Research, Lazimpat, Kathmandu 44600, Nepal; richa_np@hotmail.com; 5 Department of Global Health and Populations, Harvard T.H. Chan School of Public Health, Harvard University, Boston, MA 02115, USA; rayordeal3@gmail.com; 6 Amoha Center for Mental Health and Well-Being, Lalitpur 44600, Nepal; bijay1019@gmail.com

**Keywords:** abortion, accessibility, availability, acceptability, affordability, contraceptive, Japan, migrants, Nepal, sexual and reproductive health, unintended pregnancy

## Abstract

While all modern contraceptive methods are available for free or at minimal cost in Nepal, contraceptive devices in Japan are mainly limited to condoms, requiring Nepalese migrant women to rely on their male partners for their use. Therefore, Nepalese migrants often seek contraceptive devices from Nepal or request friends or relatives to send them from their home country. This study aimed to identify the gaps and challenges associated with Nepalese migrants’ needs for sexual and reproductive health services (SRHSs), particularly contraceptives, before and after their migration to Japan. A mixed-methods study was adopted, an explanatory sequential design (ESD) combining quantitative and qualitative approaches, and data were collected from 186 Nepalese migrants (80 females and 106 males) through an online survey and from two focus-group discussions (FGDs) conducted among 24 participants (14 females and 10 males). This study highlighted the obstacles faced by Nepalese migrants in accessing contraceptive services, such as limited options, language barriers, and high costs. The study also revealed the importance of pre-departure training in Nepal and organizing post-arrival training in Japan to increase Nepalese migrants’ awareness of the SRHSs available in Japan, thereby helping to prevent SRH-related health problems, including unintended pregnancies and abortions, in Japan.

## 1. Introduction

The Sustainable Development Goals (SDGs), specifically Goal 3 and Target 3.7, aim to ensure universal access to sexual and reproductive healthcare services (SRHSs) by 2030 [1]. This includes family planning, information, and education and the integration of reproductive health into national strategies and programs. The SDGs also recognize the positive contributions of migrants and identify them as vulnerable populations. However, little attention has been paid to migrants’ SRHS needs. More specifically, many migrant women face challenges in accessing contraceptive devices [2]. Even those who are familiar with and aware of these services and their rights in their home country often struggle to access contraceptive devices and methods due to differences in services, rules, and regulations associated with their use in their host country.

As of June 2023, according to the Ministry of Justice in Japan, Nepal was the home country of the sixth-largest foreign-born population in Japan [3]. Among 156,333 Nepalese residents, 70,310 (45%) were women, and the proportion of women has been increasing. Many were dependents (31,829), such as the wives and daughters of cooks or the owners of Indo-Nepal restaurants or the employees of companies, followed by students (21,017) and professionals (8855), who were engineers or specialists in the humanities or international services. A significant proportion of them, amounting to 61,305 (87%), were of reproductive age (15 to 49 years); this group encounters challenges in fulfilling their SRHS needs due to language barriers [4] and the limited contraceptive choices in Japan [5,6]. This can lead to unintended pregnancies and induced abortions.

The World Health Organization (WHO) lists oral contraceptives (OCs), emergency contraceptives (ECs), injectable hormonal contraceptives (Depo-Provera), and implantable contraceptives on a model list of essential medicines [7]. It has also listed abortion pills (e.g., mifepristone and misoprostol for medical abortions) as essential medicines, with the special note, “where permitted under national law and where culturally acceptable” [7]. However, the availability and strategies regarding family planning methods vary between countries; they are influenced by the country’s goals for population control and the socio-political and religious contexts, including in Japan and Nepal.

In Japan, while the Ministry of Health, Labor, and Welfare had not approved Depo-Provera and implants at the time of the survey [8], all of the options listed by the WHO are available in Nepal [9]. Differences also exist in their costs: in Japan, under the country’s policy, certain services are available with or without subsidy schemes or coverage by health insurance. OCs and ECs are not over-the-counter (OTC) medicines and require prescriptions from gynecologists. Japanese health insurance schemes cover OCs and intrauterine devices (IUDs) only for dysmenorrhea treatment, and not for contraceptive use. OCs cost approximately USD 30 per month, and ECs cost USD 60 to 180, including an initial consultation fee and a prescription fee, in addition to the cost of the medicine. Although male condoms are readily available in Japan, they are relatively more expensive than those in Nepal. Conversely, in Nepal, as family planning is a priority in the agenda of the Ministry of Health and Population, public health facilities (run by government and non-government organizations) provide all modern contraceptive items, including OCs, Depo-Provera, IUDs, implants, male and female sterilization, and male condoms, at minimal or no cost [10,11]. Moreover, abortion was legalized in 2002 [12,13], and since 2016, government clinics have been providing free and safe abortion services, along with free family planning services [14].

Owing to these challenges in accessing contraceptives (particularly female-oriented contraceptives) in Japan, Nepalese migrant women often request that their friends and relatives send contraceptives from their home country [15]. These medicines may not always be in good condition and may also be used inappropriately due to a lack of knowledge about their use among migrant women [15]. Furthermore, migrant women who used Depo-Provera or received implants in Nepal faced challenges in continuing to receive these services or remove implants in Japan. Regarding induced abortion, surgical abortion costs exceed USD 1000, and it is the only option in Japan; however, the WHO warns that it is unsafe [7], making it unaffordable, and the language barrier adds to the difficulties in accessibility for many Nepalese migrants. Moreover, psychosocial barriers render abortion unacceptable. Consequently, some women may experience unwanted pregnancies and become ill, which may even lead to abortion (complicating the situation). Furthermore, migrants must comply with Japan’s Pharmaceutical and Medical Devices Act when bringing more than two months’ worth of medication to the country [16].

Against the backdrop of the challenges regarding contraceptive-service use among Nepalese migrants, evidence of contraceptive use and the challenges faced by this population in accessing SHRS in Japan is scarce. To our knowledge, only one study has investigated the factors associated with modern contraceptive use among Nepalese migrants in Japan and its association with their quality of life (QOL) [15]. However, this study failed to explore the challenges migrant women face in accessing SRHS in Japan. The prior study did not include their male counterparts, who play a significant role in contraceptive access, and did not compare contraceptive access before and after migration. Therefore, our study investigated the contraceptives used in Nepal and after migrating to Japan and identified the gaps in accessing SRHSs—particularly contraceptives—in Japan after migration among both male and female migrants, in terms of service availability, accessibility, affordability, and acceptability (4A) framework. This framework is based on the concept of “effective coverage” [17,18].

## 2. Materials and Methods

### 2.1. Study Design and Participants

We conducted a mixed-methods study with an explanatory sequential design (ESD) that combined quantitative and qualitative approaches [19]. ESD is commonly used to bridge the gap between quantitative and qualitative data and to enhance the understanding of complex phenomena, such as health behavior. This study aimed to explore the barriers affecting contraceptive access and the reasons behind this phenomenon among the research participants. We collected the data in two stages. First, quantitative data were collected, followed by qualitative data, from Nepalese migrants aged 18 years and older, living in Japan from June 2020 to January 2021. The inclusion criteria were the same for both quantitative and qualitative data collection. Altogether, 186 individuals participated in the questionnaire survey, and 14 females and 10 males participated in two separate focus-group discussions (FGDs). They were recruited through the SNS of the research team members and snowball sampling. The qualitative data provided a foundation for the initial understanding of contraceptive access among Nepalese migrants in Japan. The quantitative data helped us to explain and contextualize the findings based on the quantitative data.

### 2.2. Data Collection and Tools

For quantitative data collection, we conducted an online questionnaire survey through a Google Form in Nepali and English languages, owing to the movement restrictions implemented due to the COVID-19 pandemic. While most participants responded to Nepali, 19 responded to English. Additionally, for 27 participants who did not have Internet access, telephone interviews were conducted by a research collaborator who read out the questions and filled in the respondents’ answers. We employed the snowball sampling method to recruit study participants for both the quantitative and qualitative data collection stages through social network services (SNSs), such as Facebook and Messenger, used by the research team members.

The authors reviewed the questionnaires used in previous studies [4,15] and finalized them after the pretests. The questionnaire survey included 34 questions separated into four sections: (A) the sociodemographic characteristics of the study participants (8 questions), including their birth year (age), sex, religion, educational background, marital status, and number of children; (B) basic information about their migration to Japan (7 questions), including their year of arrival (number of years in Japan), residential status, residential area, employment status, income, and health insurance; (C) access to SHRS in Nepal and before leaving Nepal (7 questions); and (D) access to SRHS after their arrival in Japan (12 questions). The section on access to SRHS in Nepal and Japan included items on participation in sexual and reproductive health education, use of contraceptives, and unintended pregnancies.

The participants were asked two questions about their contraceptive use in Nepal: “Which contraceptive methods did you use before migration?” and “Why didn’t you use contraceptive methods before migration?”. To inquire about their access to contraceptive methods and the types of contraceptives used after arriving in Japan, their contraceptive choices before migration, and the reasons for their choices, the following questions were asked: “Which contraceptive methods do you use in Japan?”, “Did you or your partner/spouse purchase or use any contraceptive items as preparation to come to Japan?”, and, if so, “Why did you do this?”. The reasons for not using contraceptive methods in Japan were collected using the following question: “Why don’t you use contraceptive methods in Japan?”. Regarding contraceptive access in Japan, the following question was asked: “How did you or your partner/spouse obtain contraceptive devices, condoms, or pills in Japan?”. All the items had multiple-choice responses. We categorized the responses regarding the reasons for not using contraceptives in Nepal and Japan into four groups according to the 4A framework [18]: barriers due to availability, accessibility, acceptability, and affordability.

This qualitative approach was used with the aim of gaining an in-depth understanding of SRHS utilization among participants in Nepal and after arriving in Japan. Two FDGs were also conducted virtually through Zoom among 14 female and 10 male participants, separately by sex, in Nepali, using semi-structured FGD guidelines. The guidelines were developed by the authors’ team based on previous studies [4,15] and on their experience in providing counseling to the research population as medical service providers or certified social workers. Both FGDs began with an introductory session on ensuring confidentiality to obtain oral consent, followed by a discussion focused on general access to SRH information and services in Japan, participants’ contraceptive needs and use, support of their partner or spouse, and SRHS-related challenges faced by Nepalese men and women in Japan. Questions regarding these points were frequently posed by the research population to the authors. The discussions were facilitated by male and female research team members for the male and female FGD, respectively. Both facilitators were well trained in health-behavior research, and all participants were fluent in Nepali. Therefore, linguistic and cultural barriers did not affect the data collection and the interpretation of the results. Each discussion lasted around 90 minutes and was audio-recorded. Notes were taken during the discussion.

### 2.3. Data Analysis

We adopted the 4A framework (availability, accessibility, acceptability, and affordability), namely the modified AAAQ (availability, accessibility, acceptability, and quality) framework used by Homer et al. [18], to identify the challenges faced by Nepalese migrants in accessing contraceptive services in Japan. The AAAQ model is a standard tool used to assess SRHS [18]. The following elements were included in the AAAQ:

Availability: Facilities, goods, and services must be available in sufficient quantities and in continuous supply.

Accessibility: Facilities, goods, and services must be accessible to everyone (physical access, affordability, access to information, and non-discrimination).

Acceptability: Facilities, goods, and services must be acceptable to consumers, culturally appropriate, and sensitive to vulnerable groups.

Quality: Facilities, goods, and services must be of good quality.

The AAAQ lacks a time element. Therefore, this study included “available when you want to use” to denote availability. Moreover, the quality of services cannot be analyzed through a survey. Therefore, this study focused on affordability, the financial aspect of service utilization, instead. The following factors were used for each assessment:

Availability (presence and time): Sufficient facilities, supplies, and services are available when you want to use them.

Accessibility (location and distance): Facilities, goods, services, and information that are accessible.

Affordability (cost): Facilities, goods, and services are available at affordable costs.

Acceptability (psychological aspect): Services received without language barriers or social stigma.

Adopting the ESD, we conducted a qualitative phase to explain or build upon the initial quantitative results, providing a comprehensive understanding of access to reproductive health services among Nepalese migrants in Japan. The initial collection of quantitative data helped us obtain a broader overview of the current state of access to reproductive health services among many Nepalese migrants through structured questionnaires. This allowed us to understand the situation to a certain extent in numerical terms. Later, the focus-group discussions provided deeper insights into the personal experiences, cultural barriers, and specific challenges faced by migrants that numerical data alone could not convey.

For the quantitative data, descriptive statistics, stratified by gender, generally analyzed using Excel 2021 MSO. Statistical methods, such as chi-square and Fisher’s exact tests, were not suitable for this study due to the smaller number of participants in some categories. Additionally, such tests might have led to incorrect conclusions. Therefore, the results were presented as proportions, without statistical significance tests, owing to the small sample size in certain categories. 

For qualitative data, we conducted a thorough review of the notes taken during the FGDs and audio recordings. Relevant data were extracted based on the coding framework and translated into English; they were then categorized under the four themes (availability, accessibility, acceptability, and affordability) of the 4A framework. Table 1 shows the coding framework used in this study. Important quotations were also selected, and the data were summarized. The summarized data were discussed extensively among the research-team members to draw conclusions until a consensus was reached.

### 2.4. Ethical Approval

The Sophia University Ethics Committee on Research Involving Human Subjects approved this study in June 2020. Informed consent was obtained from all participants in both the questionnaire survey and FGDs, and all data were stored in anonymized forms.

## 3. Results

### 3.1. Results from Quantitative Survey

Table 2 presents the sociodemographic and migration-related characteristics of the 186 participants. Among them, 106 (57.0%) were male, and 80 (43.0%) were female. More than half of the participants were younger than 30 years of age (51.1%), and 155 were Hindus (83.3%). Regarding education, 101 (54.3%) had a university degree or higher. Most participants were married, totaling 138 (74.2%), and 103 (54.8%) did not have children. Most of them (170) had experienced sexual intercourse (91.4%), and 143 (76.9%) had a sexual partner in Japan. In terms of migration-related characteristics, 104 (55.9%) had been in Japan for five years or less, and 75 (40.3%) had a moderately proficient level of Japanese. The most common visa status was that of an engineer/specialist in humanities/international services, exhibited by 66 respondents (35.5%), followed by student status, exhibited by 49 (26.3%). The income level ranged from JPY 100,000 to 200,000 per month for 79 respondents (42.5%), and nearly all had health insurance, i.e., 183 (98.4%).

Table 3 shows the contraceptive methods used by 140 participants (82 males and 58 females) who had sexual partners in Japan, including those who did not have sexual partners in Nepal. The table presents the responses of both male and female participants about their own and their partners’ contraceptive use. For instance, when a male participant reported Depo-Provera, it indicated his partner’s use of the method. Conversely, a female participant responded that “withdrawal” was her partner’s method of contraception. Male condoms were the most used contraceptive method among all methods reported, both in Nepal and Japan. However, female-oriented contraceptive methods, such as ECs and Depo-Provera, were primarily used in Nepal only, as reported by four male and two female participants for ECs and two male and six female participants for Depo-Provera. In Japan, only one female participant reported using ECs. As for OCs, while two male participants and one female participant responded that they used them in Nepal, three male and three female participants used them in Japan. While two participants (one male and one female) responded that they used implants in Nepal, three females responded that they used implants in Japan. Two female participants stated that they had received sterilization in Japan.

Traditional methods were also more often used in Japan than in Nepal, with 19 (23.2%) males and 15 (25.9%) females highlighting the withdrawal method, and 4 males and 11 females responding that they used the rhythm method in Japan. Regarding the non-use of contraceptives, 13 (15.9%) male and 28 (48.3%) female participants responded that they did not use any methods in Nepal, and only 10 (12.2%) males and 13 (22.4%) females did not use contraceptives in Japan.

Table 4 presents the reasons that the participants gave for not using different contraceptive methods stratified by sex and country. In Nepal, abstaining from sexual intercourse was the most commonly reported reason (four males and sixteen females); however, this reason was almost absent in Japan (one male only). Ten participants in Nepal mentioned the desire to conceive (six females and four males), and nearly the same number (nine) in Japan (six females and three males). Lack of knowledge about contraceptive methods was more frequently reported in Japan (two males and three females) than in Nepal (one male). Similarly, a lack of knowledge about how to obtain contraceptive devices was given as a reason by five participants in Japan (three males and two females) compared to two in Nepal (one male and one female). Concerns over side effects were equally reported in both countries (three males and two females each). Two female participants in Nepal mentioned opposition from a partner or spouse, but none of the male participants was in either country. The difficulty in finding the preferred contraceptive options increased from three participants (one male and two females) in Nepal to five participants in Japan (three males and two females). Lastly, cost was a barrier for one female participant in Nepal and four in Japan (two males and two females). These reasons were categorized under the four themes of availability, accessibility, affordability, and acceptability (4A framework).

Table 5 displays the types of contraceptives that the participants brought from Nepal, the services that they received before migrating to Japan, and their reasons for doing so. Condoms were the most frequently carried item, reported by thirty-five (31.8%) male and eight (10%) female participants, followed by OC pills, brought by five (4.5%) male and three (3.8%) female participants. Three (2.7%) male participants responded that they brought ECs, and one (0.9%) male and four (5%) female participants responded that they used implants before arriving in Japan. While only one (0.9%) male participant reported using intrauterine devices (IUDs), sterilization, which included vasectomy or tubectomy, was an option chosen by five (6.3%) female participants.

Sixteen (8.4%) participants brought contraceptives to Japan because they were free from their local health providers in Nepal. In addition, 15 (7.9%) responded that they were cheaper in Nepal than in Japan, and 12 (6.3%) expressed concerns about the availability of these services in Japan. The most common reason, reported by 22 (11.6%) individuals, was the ease of obtaining contraceptives in Nepal, without a language barrier. Additionally, four (2.1%) participants responded that medical professionals from Nepal had recommended them and eight (4.2%) had received suggestions from family and friends living in Japan.

Table 6 presents participants’ responses to the question evaluating their knowledge of sexual and reproductive health services in Japan. Only nine (8.5%) male and five (6.5%) female participants responded correctly to one statement, whereas the other four statements had incorrect responses.

### 3.2. Results from FGDs

The FGDs’ findings were structured under four major themes based on the 4A framework: (1) availability, (2) accessibility, (3) affordability, and (4) acceptability.

#### 3.2.1. Theme 1: Availability

Several female participants mentioned that their partners/spouses brought pills for them and that they were unaware of how they had obtained them. One female participant expressed the difficulty that her friend had faced in removing an IUD that had been inserted in Nepal.

“I do not know the details and he never told me the details about how he bought it (pills)”.

(P1 female)

“One of my friends inserted IUD back in Nepal. She wants to remove but has not been able to access health services. Any suggestions on this matter?”

(P2 female)

#### 3.2.2. Theme 2: Accessibility

Most female and male participants expressed various issues related to SRHSs in terms of the accessibility of information and services, which led to unintended pregnancies in a few cases. They pointed out language barrier as a major challenge, resulting in limited access to information and limited knowledge about the different contraceptive methods available in Japan, including a lack of correct information on emergency pill use. Moreover, some participants revealed that they relied on others to obtain contraceptives, indicating a lack of direct access.

“When I was pregnant, I went to multiple hospitals but was rejected due to the language barrier. I was very depressed at that time. Finally, I found a hospital in which doctors could communicate in English. I did pregnancy test at that hospital”.

(P2 female)

“Is there any tentative data on how many times it’s possible to use the emergency pill? Many people seem to use it impulsively. My partner used it once or twice in emergencies, and we remain concerned about whether this could lead to problems or complications in the future, such as not being able to have children. We are looking for some clarity on this issue”.

(P1 male)

#### 3.2.3. Theme 3: Affordability

Several female and male participants expressed concern regarding high costs and some services not covered by health insurance, including several SRHSs, such as abortion, emergency pills, and in vitro fertilization (IVF) with interpretation services. Some male participants were unaware of the very high costs of SRHS compared with Nepal, such as male sterilization.

“I searched for English speaking doctors and went to Shinjuku. I went to seek treatment immediately (within 2 days after sexual intercourse). He advised me to take pills for to 2–3 days. It cost 104,000 yen for that”.

(P7 female)

“Overall, I had an easy and safe abortion because I did at an early stage. I am only worried about whether it might cause any problem in conceiving a baby in the future. Moreover, the main concern is related to money. We had to pay around 150,000–200,000 yen for abortion”.

(P7 female)

“One of my sisters from Nepal wanted to do IVF in Japan. However, the hospital clearly mentioned that they should keep translators on their own if they want treatment from the hospital. Even in Tokyo, except for a few hospitals, most do not have interpreters. IVF is expensive and not covered by health insurance. The cost of IVF, along with that of the interpreter, is a huge financial burden. So, it is quite difficult”.

(P1 female)

“In Japan, how much would the cost be for male sterilization?”

(P7 male)

#### 3.2.4. Theme 4: Acceptability

Several male and female participants shared their opinions and experiences regarding the acceptability issues of certain contraceptives, their concerns about side effects, and the difficulty in making decisions about SRH due to cultural and social barriers and the stigma associated with it. One female participant mentioned a reluctance among males to use condoms, leading to negative health consequences among female partners. One male participant highlighted the culture of silence and hesitance to discuss sexual health topics, even in a confidential setting.

“I have been receiving queries from my friends who do not want to get pregnant but their partner refuse to use condom”.

(P7 female)

“There were a group of families living together. One of them conceived the baby unintentionally, immediately after delivery. They wanted to abort but were very reluctant thinking if others knew that they might spread the rumor in their hometown. They came to seek help from me. Finally, they were able to abort successfully. They were educated. The women had already completed her bachelor’s degree, and the male had been living in Japan for more than 10 years. I was sad to see our perceptions and attitudes towards abortion and reproductive health”.

(P3 female)

“It seems like everyone is shy even in confidential programs. Nobody comes forward openly, or perhaps there are no issues at all. I just cannot understand it. I’ve shared my experience and opinions though”.

(P1 male)

“We have been using condom now. I used to take pills but not anymore because I heard about its side effect from my friend”.

(P5 female)

## 4. Discussion

This mixed-methods study among Nepali migrants in Japan investigated their contraceptive use and compared the reasons for not using them in Nepal and after arriving in Japan. Our findings highlight why individuals do not use contraceptive methods and reveal the diverse and multifaceted gaps in contraceptive use in Nepal and after migrating to Japan in terms of their availability, accessibility, affordability, and acceptability (4A).

The outdated SRHS in Japan does not meet global standards. The lack of options and the high costs of contraception and abortion are common issues faced not only among migrant women but also among Japanese women. There are no statistics with which to compare contraceptive access between native Japanese and migrant women in Japan. However, migrant women face more difficulties due to restrictions in immigration laws that determine their status of residence; language barriers; and changes in the availability, accessibility, affordability, and acceptability of appropriate SRHS before and after migration. This research reveals that migration increases vulnerability, as access to SRHSs deteriorates after they reach Japan.

### 4.1. Availability

In this study, the use of male condoms remained consistently high among the participants compared to all other methods, both in Nepal and after moving to Japan, indicating the general acceptance of and preference for this method. Although female-oriented contraceptive methods, such as Depo-Provera, implants, and female sterilization, were more prevalent in Nepal [20], our survey data were quite different from the national-level statistics, showing a strong preference for condoms among our participants, even in Nepal. This might be attributed to the wide availability of condoms in Nepal, either for free or at minimal cost [10,21]. In Japan, compared to condoms, other modern contraceptive methods are neither readily available nor affordable [22], which could be the reason for the higher condom use among our participants. Our findings align with the higher prevalence of condom use in Japan, which could be since migrants tend to adapt to the contraceptive practices in their host countries [23].

In contrast to condoms, the number of study participants using female-oriented contraceptive methods, such as ECs and Depo-Provera, in Nepal decreased noticeably after their migration to Japan. This decline is likely due to ECs requiring prescription in Japan and Depo-Provera not being approved by the Japanese government, limiting its availability, which is crucial for Nepalese migrants. However, in Nepal, ECs are over-the-counter medicines that are readily available, and Depo-Provera is the most popular option among modern contraceptive methods [20]. Furthermore, while implants are another popular method in Nepal [20], only two of our study participants used this method in their home country, and three of them received it as preparation for migration to Japan. However, implants were not approved in Japan at the time of our survey, and the necessary services for their maintenance or removal are scarce, posing risks to implant users in Japan.

The two most traditional and least effective methods, namely withdrawal and the rhythm method, were also prevalent among our study participants. The number of females opting for these two methods was relatively higher in Japan than in Nepal. There could be two possible reasons for this finding: some who did not have sexual partners in Nepal started to become sexually active and began using these two traditional methods in Japan; alternatively, due to the unavailability of other modern methods, they had to rely on easily available traditional methods instead. Additionally, we observed a decrease in the number of individuals who did not use contraceptive methods after migrating to Japan, indicating a potential shift in their contraceptive practices post-migration. A significant reason for the non-use of contraceptives in Nepal was abstinence from sexual intercourse, particularly among females. However, this reason has not been reported in Japan; this could possibly reflect a change in lifestyle or relationship status due to the more open society in Japan compared to Nepal. This may lead female migrants to experience adverse consequences such as unintended pregnancies and abortions.

Our findings imply that the lack of contraceptive methods, readily available in Nepal but restricted by different policies in Japan, can be a major challenge for Nepalese migrants. This disparity can influence their decisions in choosing contraceptive methods, which may not always be the most suitable option.

### 4.2. Accessibility

Of the several reasons that our participants gave for avoiding contraceptive methods in Nepal and Japan, two were “did not know how to get contraceptive devices” and “did not know about contraceptive methods”. This indicates a lack of adequate knowledge of contraceptives and limited access to information about these devices and methods. Moreover, only a small number of participants had accurate knowledge of the various SHRSs in Japan, which could be associated with decreased contraceptive use [15]. One possible reason for the limited knowledge of contraceptives and their accessibility among our study participants could be the language barrier. More than 60% of the participants had only a low or medium level of Japanese-language skills, equal to the daily conversational level of Japanese or below.

Furthermore, 65 participants in this study either brought some form of contraceptive from Nepal or received it while migrating to Japan, and 22 of them cited “easy to obtain (without language barriers)” as one of the reasons for doing so. Many participants of both sexes in the FGDs mentioned that language was a significant challenge for accessing SRH information and services. Previous studies have also reported insufficient language skills as a barrier to contraceptive use among migrant populations in Japan [4,15]. This language barrier may lead migrants to bring contraceptives from Nepal or ask their family members or friends to send them to Japan. Some female participants in the FGD mentioned that they received pills from their partners and were not informed about how their partners had received them because prescriptions are required for pills in Japan. A previous study of Nepalese migrants reported similar findings [15]. The improper and illegal procurement of contraceptive medicines may lead to their abuse, resulting in negative health consequences.

### 4.3. Affordability

Affordability was cited as one of the reasons for the non-use of contraceptives in Japan by four study participants who found them “too expensive to use”. This suggests that contraceptive devices and medications may not be financially accessible for some individuals. Regarding their reasons for preparing contraceptive items before migration, 16 participants responded that they were “free at a local provider in Nepal”, while another 15 responded that they were “available at a lower price in Nepal than that of Japan”. Additionally, the participants in the FGDs expressed concerns regarding the high cost of some SHRS and those not covered by insurance. For example, abortion, which is a fundamental reproductive health right, can cost JPY 150,000 to 200,000, and one female participant shared an experience in which she paid JPY 104,000 for EC pills from an English-speaking doctor. This price could have been unaffordable for many of our study participants, whose monthly incomes ranged between JPY 100,000 and 200,000 (Table 2). Affordability is a significant barrier to regular contraceptive use among Nepalese migrants in Japan. While many participants reported purchasing condoms or accessing other contraceptive services at pharmacies or other medical institutions in Japan, these services are often free or available at a minimal cost in Nepal. Previous studies on Nepalese and Burmese migrants in Japan also revealed similar issues regarding affordability [4,15].

### 4.4. Acceptability

In terms of acceptability, this study identified several barriers to contraceptive use among our study participants. Concerns about the side effects were reported by five participants in Nepal and Japan. This concern was also expressed by both male and female participants in FGDs. The reluctance of male participants to use condoms was another issue related to acceptability. Although only two female participants reported this in the quantitative survey, one female participant in the FGD mentioned that several of her friends shared this concern with her. Husbands’ opposition to the use of condoms has been cited as a challenge to contraceptive use in Japan [15]. Furthermore, one female participant discussed the cultural and social stigma associated with abortion. Additionally, some male and female participants highlighted the culture of silence and hesitance to discuss contraceptive use and sexual health topics, even in settings that assured confidentiality. Such cultural norms can significantly impact the acceptability of contraceptive use, as reported in a previous study [4].

### 4.5. Limitations

Our study’s findings should be interpreted with consideration of several limitations. First, although confidentiality was assured in both the quantitative survey and FGDs, some respondents might have been embarrassed to share information about their sex lives and might have presented socially acceptable responses, which could have led to social-desirability bias.

Second, because this study was conducted during the COVID-19 pandemic, it faced several restrictions. Cross-border movement restrictions led to the cancellation of international flights, making it challenging to obtain OCs by postal mail or through relatives or friends who bought them in Nepal. This point should have been included in the questionnaire and FGDs, although only a few might have responded to this question. The movement restrictions within Japan could have led to the limited number of participants in both the quantitative survey and FGDs because of the lack of in-person events and gatherings to share information among migrants. This also limited our analysis to descriptive statistics. Additionally, the views expressed in the FGDs might not have captured participants’ diverse perspectives, and a comprehensive understanding of the topic might not have been reached. Thus, our findings may not be representative of all Nepalese migrants living in Japan. Nevertheless, our study included male participants and presented their insights and perspectives, which were not included in previous studies of the migrant population in Japan [4,15].

Third, the use of an online survey tool was not ideal for Nepalese migrants with limited Internet access or for those who were unfamiliar with digital tools. This could also have led to the limited participation and disproportionate representation of Nepalese migrants in Japan regarding their education levels, income levels, and residential status. This might also have prevented us from reaching the most vulnerable groups facing comparatively more challenges. The limited number of participants in the quantitative survey and the use of only two FGDs might have led to the underestimation of the magnitude of SRH problems faced by Nepalese migrants in Japan.

## 5. Conclusions

This mixed-methods study among Nepalese migrants in Japan provided insights into the status of their contraceptive use and identified gaps in their access to these services. Despite the preference for condoms among our study participants, this study uncovered several challenges, such as the limited availability of contraceptive choices; the absence of certain services; language barriers; a lack of knowledge and access to information; high costs that limit the affordability of certain services/methods; and the acceptability of some methods due to psychological, cultural, and social norms. Our findings underscore the importance of incorporating SRH education into pre-departure training in Nepal (through language schools, academic consultants, and recruitment agencies) and organizing post-arrival training in Japan (through schools, companies, and recruitment agencies). For instance, Comprehensive Sexuality Education, promoted by UNESCO, can be modified for migrants to equip them with knowledge, skills, attitudes, and values that enable them to consider their life plans and to ensure the protection of their rights. This would increase Nepalese migrants’ awareness of contraceptives and SRH services available in Japan, thus helping prevent SRH-related health problems, including unintended pregnancies and induced abortions. Further research with a broader representation of Nepalese migrant community is essential for a better understanding of their SRHS needs in Japan.

## Figures and Tables

**Table 1:** Coding framework used to study access to contraceptive services among Nepalese migrants in Japan.

Key Theme	Description
Availability	
Unavailability	Preferred option unavailable in Japan so difficulty to obtain the certain methods
Easy availability	Several options easily available in Nepal compared to Japan
Difficulty to obtain	Not easily available; need to obtain from different sources (from family and friends)
Accessibility	
To information	Difficulty in access to contraceptives due to lack of knowledge about contraceptives
To services	Difficulty in access to contraceptives due to lack of information on how to get them, lack of direct access
Language barrier	Difficulty in access to contraceptives due to language barrier
Affordability	
High costs	Very high costs of several contraceptive services in Japan
Insurance	SHRH services not covered by insurance in Japan
Free of cost/cheap	Most of the contraceptives in Nepal are available for free or at a minimal cost
Acceptability	
Opposed by spouse/partner	Spouse/partner deny using contraceptives
Cultural and social barriers	People are generally shy about talking sexual health issues; abortion is not accepted in the society
Fear about side effects	Concerned over side effects of certain types of contraceptives due to lack of information

**Table 2:** Sociodemographic and migration characteristics of participants (male, 106; female, 80; and total, 186).

Variable	Male n (%)	Female n (%)	Total n (%)
Age (years)			
≥30	55 (51.9)	35 (43.8)	90 (48.4)
≤29	51 (48.1)	44 (55.0)	95 (51.1)
No response	0 (0.0)	1 (1.3)	1 (0.5)
Religion			
Hindu	90 (84.9)	65 (81.3)	155 (83.3)
Other/none	16 (15.1)	15 (18.8)	31 (16.7)
Education			
High school and below (12 years of education)	53 (50.0)	32 (40.0)	85 (45.7)
University or above	53 (50.0)	48 (60.0)	101 (54.3)
Marital Status			
Married with a spouse from your country or different country	79 (74.5)	59 (73.8)	138 (74.2)
Single, divorced, separated, or widowed	27 (25.5)	21 (26.3)	48 (25.8)
Child/children			
No	62 (58.5)	40 (50.0)	102 (54.8)
Yes	44 (41.5)	40 (50.0)	84 (45.2)
Experience of sexual intercourse			
Yes	100 (94.3)	70 (87.5)	170 (91.4)
No	6 (5.7)	10 (12.5)	16 (8.6)
Ever had a sexual partner in Japan			
Yes	82 (77.4)	61 (76.3)	143 (76.9)
No	24 (22.6);	18 (22.5)	42 (22.6)
No response	0 (0.0)	1 (1.3)	1 (0.5)
Years in Japan (years)			
≤5	54 (50.9)	50 (62.5)	104 (55.9)
6–10	44 (41.5)	17 (21.3)	61 (32.8)
>10	8 (7.5)	12 (15.0)	20 (10.8)
No response	0 (0.0)	1 (1.3)	1 (0.5)
Japanese language level			
Low	16 (15.1)	25 (31.3)	41 (22.1)
Medium	45 (42.5)	30 (37.5)	75 (40.3)
High	45 (42.5)	25 (31.3)	70 (37.6)
Visa status			
Student	26 (24.5)	23 (28.8)	49 (26.3)
Dependent	5 (4.7)	35 (43.8)	40 (21.5)
Engineer/specialist in humanities/international services	57 (53.8)	9 (11.3)	66 (35.5)
Permanent resident/long-term resident/other	18 (17.0)	13 (16.3)	31 (16.7)
Income			
None	7 (6.6)	8 (10.0)	15 (8.1)
Less than JPY 100,000	16 (15.1)	37 (46.3)	53 (28.5)
JPY 100,000–200,000	53 (50.0)	26 (32.5)	79 (42.5)
Over JPY 200,000	30 (28.3)	9 (11.3)	39 (20.9)
Health insurance			
Paying by myself	63 (59.4)	33 (41.3)	96 (51.6)
Being paid by family	2 (1.9)	36 (45.0)	38 (20.4)
Being paid by employer	38 (35.8)	11 (13.8)	49 (26.4)
Stopped paying/never paid	3 (2.8)	0 (0.0)	3 (1.6)

**Table 3:** Contraceptive methods used in Nepal and Japan (male, 82; female, 58; and total, 140).

Contraceptive Method	Male n (%)		Female n (%)	
	Nepal	Japan	Nepal	Japan
Modern methods				
Male condom	65 (79.3)	62 (75.6)	20 (34.5)	23 (39.7)
Oral contraceptive (OC)	2 (2.4)	3 (3.7	1 (1.7)	3 (5.2)
Emergency contraceptive (EC)	4 (4.9)	0 (0.0)	2 (3.4)	1 (1.7)
Injectable hormonal contraceptive (Depo-Provera)	2 (2.4)	0 (0.0)	6 (10.3)	0 (0.0)
Implantable contraceptive (implant)	1 (1.2)	0 (0.0)	1 (1.7)	3 (5.2)
Intrauterine device (IUD)	0 (0.0)	1 (1.2)	0 (0.0)	0 (0.0)
Sterilization	0 (0.0)	0 (0.0)	0 (0.0)	2 (3.4)
Traditional methods				
Withdrawal	20 (24.4)	19 (23.2)	8 (13.8)	15 (25.9)
Rhythm method	7 (8.5)	4 (4.9)	2 (3.4)	11 (19.0)
None	13 (15.9)	10 (12.2)	28 (48.3)	13 (22.4)

**Table 4:** Reasons for not using contraceptive methods in Nepal and Japan.

Reason (4A Framework)	Male (Nepal, 13; Japan, 10)		Female (Nepal, 28; Japan, 12)	
	Nepal n (%)	Japan n (%)	Nepal n (%)	Japan n (%)
Not having sexual intercourse	4 (30.8)	1 (10.0)	16 (57.1)	0 (0.0)
Wanted to get pregnant	4 (30.8)	3 (30.0)	6 (21.4)	6 (50.0)
Concerned over side effects	3 (23.1)	3 (30.0)	2 (7.1)	2 (16.7)
Could not find own preferred options (availability)	1 (7.7)	3 (30.0)	2 (7.1)	2 (16.7)
Did not know how to get contraceptive devices (accessibility)	1 (7.7)	3 (30.0)	1 (3.6)	2 (16.7)
Did not know contraceptive methods (accessibility)	1 (7.7)	2 (20.0)	0 (0.0)	3 (25.0)
Too expensive to use (affordability)	0 (0.0)	2 (20.0)	1 (3.6)	2 (16.7)
Opposed by partner/spouse (acceptability)	0 (0.0)	0 (0.0)	2 (7.1)	0 (0.0)

**Table 5:** Types of contraceptives chosen in Nepal or brought to Japan, reasons for their selection, or reasons for bringing them to Japan (male, 110; female, 80; and total, 190).

Types of Contraceptives Chosen in Nepal or Brought to Japan	Male (%)	Female (%)	Total (%)
None	64 (58.2)	59 (73.8)	123 (64.7)
Condom	35 (31.8)	8 (10.0)	43 (22.6)
Oral contraceptive pill	5 (4.5)	3 (3.8)	8 (4.2)
Emergency contraceptive pill	3 (2.7)	0 (0.0)	3 (1.6)
Implant/Norplant	1 (0.9)	4 (5.0)	5 (2.6)
Intrauterine device (IUD)	1 (0.9)	0 (0.0)	1 (0.5)
Sterilization (vasectomy/tubectomy)	0 (0.0)	5 (6.3)	5 (2.6)
Reasons for selection or bringing them to Japan			
Free at local health provider in Nepal	12 (10.9)	4 (5.0)	16 (8.4)
Available at a cheaper price than Japan	11 (10.0	4 (5.0)	15 (7.9)
Worried about available service in Japan	8 (7.3)	4 (5.0)	12 (6.3)
Easily to obtain (without language barrier)	15 (13.6)	7 (8.8)	22 (11.6)
Suggestions by local medical professionals from Nepal	2 (1.8)	2 (2.5)	4 (2.1)
Suggestions by family and friends living in Japan	1 (0.9)	7 (8.8)	8 (4.2)

**Table 6:** Knowledge of sexual and reproductive health services in Japan (multiple-choice answers; 106 men and 77 women).

Statement	Answer	Male n (%)	Female n (%)
Surgery is the only option for abortion in Japan (at the time of the survey)	Correct	9 (8.5)	5 (6.5)
Rhythm method and withdrawal are enough to prevent pregnancy	Wrong	67 (63.2)	37 (48.1)
Migrants can bring enough contraceptive pills for a year from own country	Wrong	9 (8.5)	14 (18.2)
Anyone can purchase EC at pharmacies without prescription	Wrong	11 (10.4)	2 (2.6)
None of the above is true	Wrong	24 (22.6)	24 (31.2)
No answer		3 (2.8)	4 (5.2)

## Data Availability

Data are contained within the article.
